# 1984—Discovery of the First Anti-HIV Drug, Suramin

**DOI:** 10.3390/v13081646

**Published:** 2021-08-19

**Authors:** Erik De Clercq

**Affiliations:** Rega Institute for Medical Research, Department of Microbiology, Immunology and Transplantation, KU Leuven, Herestraat 49, B-3000 Leuven, Belgium; erik.declercq@kuleuven.be

## 1. Introduction

In 2021, we commemorate the 40th anniversary of the identification of the disease AIDS, the acquired immune deficiency syndrome, a name that for the first time in history was launched in 1981. The etiologic origin of the disease was tentatively assigned to the LAV-1 (lymphadenopathy virus type 1)/HTLV-III (human T-lymphocytic virus type 3) in 1983 [1,2] (the name HIV-1 (human immunodeficiency virus type 1 was introduced in 1987 to replace previous names used for this virus).

While it was widely anticipated that it would be feasible to develop an effective vaccine for this disease, such a vaccine, even 40 years after it was first announced, is still not forthcoming. Instead, a wealth of anti-HIV drugs have been formally approved by the US FDA for the treatment of HIV-1 infections, and two of them (Truvada^®^ and Descovy^®^) have been licensed for the prevention (PrEP (Pre-Exposure Prophylaxis)) of HIV infections.

This situation is in sharp contrast with the current COVID-19 (SARS-CoV-2) pandemic, which can now be prevented by an increasing number of vaccines, and for which, at present, only one antiviral drug, Remdesivir (Veklury^®^), has been formally approved by the US FDA.

## 2. Discovery of the Reverse Transcriptase (RT)

After having spent (as a post-doctoral researcher) 2 years (1969–1970) at the Stanford University Medical School, where my research work was primarily focused on the mechanism of interferon induction by synthetic polyanions, I had to return back home, not without having read two intriguing articles in Nature [3,4], both pointing to the existence of an enzyme (RT) that was able to (reversely) transcribe RNA to DNA. Not more than 5 years later, in 1975, Howard Temin and David Baltimore, together with Renato Dulbecco, would be honored with the Nobel Prize for Physiology and Medicine.

As soon as I was settled back in my laboratory in the Rega Institute, in 1971, I was extremely anxious to find out whether MLV (Moloney Murine leukemia virus), as described by Temin and Baltimore, indeed contained such a kind of enzyme (RT), which I verified by measuring the radioactivity counts of the trichloroacetic acid (TCA) precipitated DNA product(s) labeled with radioactive tritium (^3^H). It is hard to explain how exhilarated I was when confirming the data of Temin and Baltimore.

The availability of an RT assay would now allow me to search for any compounds that would be able to inhibit the RT activity and thus be capable of suppressing any processes in which the RT would play a dominant role.

The RT assay I had developed was then used to evaluate several compounds as potential RT inhibitors. A typical example is represented by the gliotoxin analogues which were reported in 1978 [5,6,7].

Also evaluated (around 1975) against the MLV RT was suramin hexasodium salt (Moranyl^®^) obtained from Rhône-Poulenc (Société Parisienne d’Expansion Chimique, Paris, France) (Suramin is known as Antrypol, Bayer 205, Germanin, Moranyl, Naganol, Naganin, or Naphuride sodium and has been used since the 1920s in the therapy and prophylaxis of African trypanosomiasis (sleeping sickness)). It was found to be a more potent RT inhibitor than the gliotoxin analogues [5,6,7] and several other compounds, including ammonium 5-tungsto-2-antimoniate [8], when evaluated under the same conditions.

In the 1970s, the possible role of RT in the pathogenesis of cancer became a real hype after Sol Spiegelman had first reported the presence of RT in various virions including visna [9], and those isolated from mammary carcinoma [10,11], and murine leukemia [12]. It was further reported in a dazzling number of papers in PNAS [13,14,15,16,17,18], that all sorts of human cancer (breast cancer, leukemia, Burkitt’s lymphoma, brain tumors, Hodgkin’s lymphoma, gastrointestinal and lung malignancies, and malignant breast tumors) displayed enhanced RT levels.

Incited by these claims, I started to verify whether suramin could exhibit some inhibitory activity (based on mortality scores) in mice which had been inoculated with (murine) leukemic cells. Unfortunately, I did not witness any reduction of the mortality rates in mice given suramin.

In 1978, Prof. R.C. Gallo visited us at the Rega Institute, and when I had informed him about my experience(s) with suramin, he found that my data obtained with the effects of suramin on the RT of RNA tumor viruses were worth publishing, and hence, the paper on “Suramin: a potent inhibitor of the reverse transcriptase of RNA tumor viruses” appeared in 1979 in Cancer Letters [19] (it is one of the few of my papers that did not need revision before publication).

Years went by and I no longer expected that suramin would generate any interest until in 1984 (apparently on 11 September or shortly thereafter) I received a phone call from Sam Broder, NCI (National Cancer Institute) revealing that based on the 1979 paper in Cancer Letters [19] he and his colleagues (including R.C. Gallo) had evaluated suramin and found it to be effective in inhibiting HTLV-III replication in cell culture [20].

The following year, Sam Broder and his colleagues (now including Anthony Fauci besides R.C. Gallo) reported that suramin would also prove inhibitory to the replication of HTLV-III/LAV in vivo, in patients presenting as Kaposi’s sarcoma or AIDS-related complex [21].

On 2 June 1985 a confidential meeting was organized at NIH (NCI), where Sam Broder would divulge the present situation in the search for drug treatment of AIDS. I had the privilege to have been invited to attend without being a speaker. Sam Broder reviewed the current situation on suramin, then revealing they (at NCI) had come across a new compound, apparently received from Burroughs Wellcome, with the code number of BW A509U, that was even more potent and less toxic than suramin in inhibiting the in vitro replication of HTLV III/LAV. As representative of the Wellcome Laboratories at Research Triangle Park, David W. Barry was also present. All meeting attendants were deeply curious to know the chemical structure of BW A509U. Dave Barry replied he knew but was not going to tell it, only that “it was a nucleoside analogue but not acyclovir”. We had to wait till October 1985, for the PNAS paper on BW A509U to be published. Then, the whole community learned it was AZT (3′-azido-3′-deoxythymidine) [22], the nucleoside that would herald the advent of a broad range of 2′,3′-dideoxynucleoside analogues later to be extended to ddI, ddC, d4T, 3TC, ABC, (-)FTC, and now collectively referred to as NRTIs (“Nucleoside Reverse Transcriptase Inhibitors”).

On 23 February 1985, a few months after the Science paper on suramin had appeared (12 October 1984), a letter in The Lancet appeared on HPA 23, in the treatment of three patients with AIDS and one with prodrome [23]. HPA 23 was identified as ammonium 21-tungsto-9-antimoniate and must have been identical or similar to the compound described by Chermann et al. in 1975 [8]. As published in The Lancet paper [23] the use of HPA 23 was associated with virus reduction as assessed by RT activity. Dormont et al. [24] further ascertained that HPA23 or ammonium-21-tungsto-9-antimoniate indeed inhibited the RT of the AIDS retrovirus.

Although the rationale of evaluating and discovering suramin as an inhibitor of HIV replication was based upon its inhibitory activity against the viral RT, its anti-HIV activity may have resulted from interference of a multitude of proteins and enzymes [25], albeit resulting in suppression of the virus replication at concentrations that were non-toxic to the host cells.

Suramin laid the basis for the identifications of various other polyanionic substances, such as aurintricarboxylic acid and Evans Blue, that at least in part owed their inhibitory effect on HIV replication to interference with the viral RT [26,27,28].

**Figure 1:**
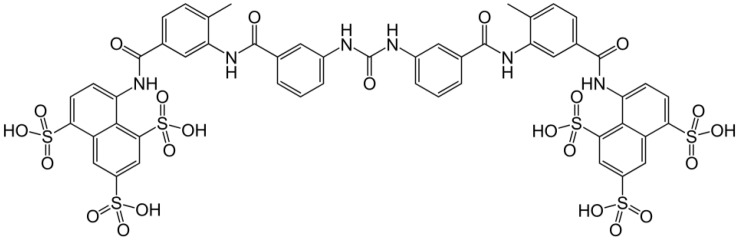
Molecular structure of Suramin. Suramin is a poly(hexa)sulfonate (hexa)sodium salt.

I suggested that polyanionic substances are potential anti-HIV agents because of their putative effect on the virus adsorption process [29]. Ito et al. [30] and Ueno and Kuno [31] then reported that sulfated polysaccharides such as dextran sulfate and heparin were highly selective inhibitors of HIV replication in vitro. They were found to inhibit virus adsorption to the host cells [32]. For dextran sulfate, Mitsuya et al. [33] reached the same conclusion. In their report [33], they referred to suramin as “a compound with six sulfate groups”. It should have been six sulfonate groups (Figure 1).
